# High carriage of adherent invasive *E. coli* in wildlife and healthy individuals

**DOI:** 10.1186/s13099-018-0248-7

**Published:** 2018-06-14

**Authors:** Oumaïra Rahmouni, Cécile Vignal, Marie Titécat, Benoît Foligné, Benjamin Pariente, Laurent Dubuquoy, Pierre Desreumaux, Christel Neut

**Affiliations:** 1 Lille Inflammation Research International Center, UMR 995 Inserm, Lille University, CHRU Lille, Lille, France; 2 Centre de Biologie Pathologie Génétique, CHU Lille, Lille, France; 3 Service des Maladies de l’Appareil Digestif et de la Nutrition, Hôpital Claude Huriez, CHU Lille, 59037 Lille, France; 4 Laboratoire de Bactériologie, 3, Rue de Pr. Laguesse, B.P. 83, 59006 Lille Cedex, France

**Keywords:** Adherent invasive *Escherichia coli* (AIEC), Healthy individuals, ECOR collection, Pathobiont

## Abstract

**Background:**

Adherent invasive *Escherichia coli* (AIEC) are suspected to be involved in the pathogenesis of inflammatory bowel diseases. Since AIEC was first described in 1999, despite important progress on its genomic and immune characterizations, some crucial questions remain unanswered, such as whether there exists a natural reservoir, or whether there is asymptomatic carriage. The ECOR collection, including *E. coli* strains isolated mainly from the gut of healthy humans and animals, constitutes an ideal tool to investigate AIEC prevalence in healthy condition. A total of 61 *E. coli* strains were examined for characteristics of AIEC.

**Methods:**

The adhesion, invasion and intramacrophage replication capabilities (AIEC phenotype) of 61 intestinal *E. coli* strains were determined. The absence of virulence-associated diarrheagenic *E. coli* pathotypes (EPEC, ETEC, EIEC, EHEC, DAEC, EAEC), and uropathogenic *E. coli* was checked.

**Results:**

Out of 61 intestinal strains, 13 (21%) exhibit the AIEC phenotype, 7 are from human origin and 6 are from animal origin. Prevalence of AIEC strains is about 24 and 19% in healthy humans and animals respectively. These strains are highly genetically diverse as they are distributed among the main described phylogroups. Among *E. coli* strains from the ECOR collection, we also detected strains able to detach I-407 cells.

**Conclusions:**

Our study described for the first time AIEC strains isolated from the feces of healthy humans and animals.

**Electronic supplementary material:**

The online version of this article (10.1186/s13099-018-0248-7) contains supplementary material, which is available to authorized users.

## Background

Crohn’s disease (CD), one of the clinical presentation of inflammatory bowel diseases (IBD), is characterized by chronic lesions of varying intensity along the gastrointestinal tract resulting from an exacerbated reaction of a defective immune system [1–3]. The etiology of CD is complex and is multifactorial, one of these factors is the state of the intestinal microbiota [4]. Over the last 20 years, *Escherichia coli* has attracted the most attention in respect to CD etiology. A high mucosal colonization level by *E. coli* was first demonstrated in CD [5]. A new pathogenic group of *E. coli*, called adherent invasive *E. coli* (AIEC), harboring adhesive and invasive abilities to intestinal epithelial cells, was described [6–8]. The main characteristics of AIEC are (i) the ability to adhere to and invade intestinal epithelial cells, (ii) the ability to survive and replicate expansively within macrophages without triggering host cell death, and (iii) the lack of known invasive determinants [10].

The AIEC strains isolated to date are clonally diverse and belong to distinct serotypes [9–11]. The B2 phylogroup is most prevalent, but strains of A, B1, and D phylogroups have also been isolated [8, 10, 12, 13]. AIEC strains have virulence factors in common with other pathogenic *E. coli* and are closely related to extra-intestinal pathogenic *E. coli* (ExPEC), associated with urinary tract infections and neonatal meningitis [14, 15]. In absence of common and specific genetic signatures between AIEC strains, this pathotype can up to now only be identified by phenotypical traits. AIEC have been described in other human intestinal disorders such as ulcerative colitis (UC), another form of IBD, and colorectal cancer but also in animals with intestinal diseases [16–18]. However, AIEC carriage is not restricted to intestinal inflammatory conditions and AIEC have also been found in biopsies of patients with functional intestinal disorders and in asymptomatic subjects undergoing surveillance colonoscopy, subjects termed as “healthy” in AIEC prevalence studies [6, 9, 12, 13, 16]. A better knowledge of AIEC carriage in healthy condition whether in human or animals will help in understanding more about AIEC natural reservoir and transmission.

The aim of this work was to determine if AIEC is present in the *E. coli* Reference (ECOR) Collection. The ECOR collection is a set of *E. coli* strains of natural origin, isolated from the gastrointestinal tract of healthy humans and non-human mammalians from a variety of geographic locations, and designed to represent the variation and genetic structure of *E. coli* in natural populations [19]. 61 ECOR strains were isolated from feces of healthy hosts (Table 1), making this collection an ideal tool to study the prevalence of AIEC in healthy conditions.

**Table 1:** Fecal strains investigated in this study

ID	Serotype	Phylogroup	Source			ID	Serotype	Phylogroup	Source		
			Host	Sex	Location				Host	Sex	Location
ECOR1	ON:HN	A	Human	Woman	USA	ECOR34	O88:NM	B1	Dog		USA
ECOR2	ON:H32	A	Human	Man	USA	ECOR35	O1:NM	D	Human	Man	USA
ECOR3	O1:NM	A	Dog		USA	ECOR36	O79:H25	D	Human	Woman	USA
ECOR4	ON:HN	A	Human	Woman	USA	ECOR37	ON:HN	E	Marmoset		USA
ECOR5	O79:NM	A	Human	Woman	USA	ECOR38	O7:NM	D	Human	Woman	USA
ECOR6	ON:NM	A	Human	Man	USA	ECOR39	O7:NM	D	Human	Woman	Sweden
ECOR7	O85:HN	A	Orangutan		USA	ECOR41	O7:NM	D	Human	Man	Tonga
ECOR8	O86:HN	A	Human	Woman	USA	ECOR42	ON:H26	E	Human	Man	USA
ECOR9	ON:NM	A	Human	Woman	Sweden	ECOR43	ON:HN	E	Human	Woman	Sweden
ECOR10	O6:H10	A	Human	Woman	USA	ECOR44	ON:HN	D	Cougar		USA
ECOR12	O7:H32	A	Human	Woman	Sweden	ECOR45	ON:HM	B1	Pig		Indonesia
ECOR13	ON:HN	A	Human	Woman	Sweden	ECOR46	O1:H6	D	Celebese ape		USA
ECOR15	O25:NM	A	Human	Woman	Sweden	ECOR47	OM:H18	D	Sheep		New Guinea
ECOR16	ON:H10	A	Leopard		USA	ECOR49	O2:NM	D	Human	Woman	Sweden
ECOR17	O106:NM	A	Pig		Indonesia	ECOR51	O25:HN	B2	Human infant		USA
ECOR18	O5:NM	A	Celebese ape		USA	ECOR52	O25:H1	B2	Orangutan		USA
ECOR19	O5:HN	A	Celebese ape		USA	ECOR53	O4:HN	B2	Human	Woman	USA
ECOR20	O89:HN	A	Steer		Bali	ECOR54	O25:H1	B2	Human		USA
ECOR21	O121:HN	A	Steer		Bali	ECOR56	O6:H1	B2	Human	Woman	Sweden
ECOR22	ON:HN	A	Steer		Bali	ECOR57	ON:NM	B2	Gorilla		USA
ECOR23	O86:H43	A	Elephant		USA	ECOR58	O112:H8	B1	Lion		USA
ECOR24	O15:NM	A	Human	Woman	Sweden	ECOR59	O4:H40	B2	Human	Man	USA
ECOR25	ON:HN	A	Dog		USA	ECOR61	O2:NM	B2	Human	Woman	Sweden
ECOR26	O104:H21	B1	Human infant		USA	ECOR63	ON:NM	B2	Human	Woman	Sweden
ECOR27	O104:NM	B1	Giraffe		USA	ECOR65	ON:H10	B2	Celebese ape		USA
ECOR28	O104:NM	B1	Human	Woman	USA	ECOR66	O4:H40	B2	Celebese ape		USA
ECOR29	O150:H21	B1	Kangaroo rat		USA	ECOR67	O4:H43	B1	Goat		Indonesia
ECOR30	O113:H21	B1	Bison		Canada	ECOR68	ON:NM	B1	Giraffe		USA
ECOR31	O79:H43	E	Leopard		USA	ECOR69	ON:NM	B1	Celebese ape		USA
ECOR32	O7:H21	B1	Giraffe		USA	ECOR70	O78:NM	B1	Gorilla		USA
ECOR33	O7:H21	B1	Sheep		USA						

This table was build according to data from previously published papers [19, 20]

## Results

### Adherent and invasive capacity of ECOR strains isolated from the feces of healthy individuals

The ECOR collection contains 61 strains of fecal origin. Of these, 29 were from human and 32 from animals (Table 1). An invasion assay was performed on these strains using I-407 cells based on gentamicin protection, according to previously described methods [6, 15, 21, 22]. All of the strains were first tested for their susceptibility to gentamicin using disc diffusion method as only strains susceptible to gentamicin can be tested. All the strains were found to be susceptible to gentamicin, determined as a zone diameter superior to 17 mm, according to EUCAST recommendations.

The invasion levels of the noninvasive references *E. coli* strain K-12 C600 and *E. coli* Nissle were 0.0561 ± 0.0373% and 0.0254 ± 0.0265% respectively. Reference strain LF82, included in all of the assays as a positive invasive control, gave a mean invasion level of 1.0101 ± 0.9880%. Another positive invasive control *E. coli* NRG857c showed a mean invasion of 0.1683 ± 0.0368%. The invasion levels were done in triplicate.

**Fig. 1:**
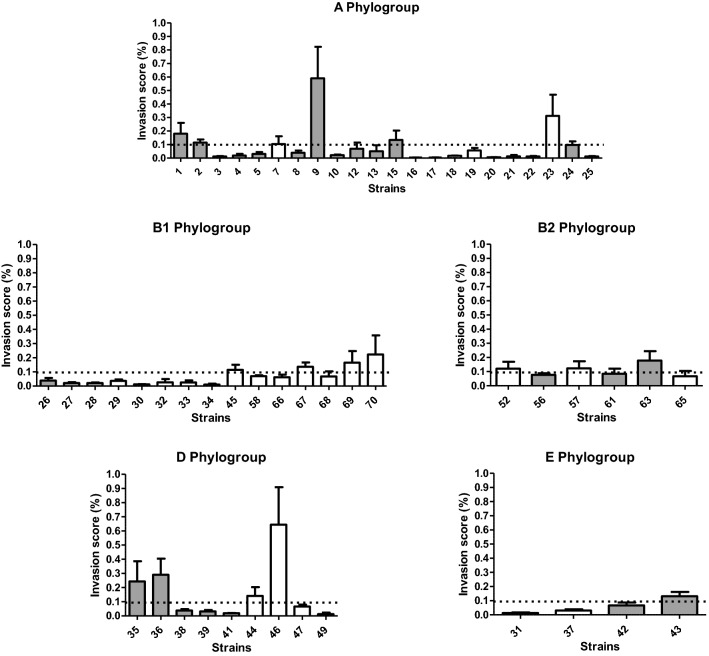
Invasion score of ECOR strains of fecal origin according to their phylogroup (excluding the cell-detaching strains)

Among strains belonging to the ECOR collection, 18 strains (30%) were classified as invasive based on this assay, with invasion rates ranging between 0.10 and 0.64%. Eight of these were of human origin (28%), four belonged to the phylogroup A (ECOR 1, 2, 9 and 15), one to phylogroup B2 (ECOR 63), two to phylogroup D (ECOR 35 and ECOR 36) and one to the phylogroup E (ECOR 43). Ten were of animal origin (31%), two belonging to the phylogroup A (ECOR 7 and 23), four to the phylogroup B1 (ECOR 45, 67, 69 and 70), two to the phylogroup B2 (ECOR 52 and 57) and two to the phylogroup D (ECOR 44 and 46) (Fig. 1).

During the invasion assay, for five of the strains, an unexpected pathogenic feature was discovered: the ability to detach intestinal cells from the microplate. All of these strains were of human origin, one belonging to the phylogroup A (ECOR 6) and the 4 others to the phylogroup B2 (ECOR 51, 53, 54 and 59) (Table 3). Invasion scores of these cell-detaching strains were established but were not interpretable due to their capacity to detach adherent cells from the microplate. The actual concentration of the intracellular bacteria was therefore skewed. Further investigations are necessary to define their propensity to invade.

The strains revealed as invasive were tested for their ability to adhere. All the invasive strains from the ECOR collection were adherent, with an adhesion index ranging between 67 and 334, all showed an adhesion index superior to 1 bacteria per cell (Table 3).

### Virulence genotyping of the invasive strains and cell-detaching strains

The 18 fecal strains previously classified as adherent and invasive were genotyped. The presence of 12 selected virulence genes associated with diarrheagenic *E. coli* pathotype was assessed. This included common virulence factors present in diarrheagenic *E. coli* pathotypes EHEC (*stx*-*1*, *stx*-*2*, *eae* and *ehxA*), EIEC (*ipaC*), ETEC (*estA*, *elt* and *tia*), EPEC (*eae*, *ehxA*, *bfpA*), EAEC (*aggR* and pCVD432) and DAEC (*afaD*) (Additional file 1: Table S1). Three strains were positive for the *tia* gene (ECOR 57) and *tia* and *afaD* genes (ECOR 63 and 70). These strains are not affiliated as AIEC pathovars and belong to ETEC/DAEC pathovars. The other 15 invasive strains (ECOR 1, 2, 9, 15, 35, 36, 43, 7, 23, 45, 67, 69, 52, 44, 46) were negative for the tested virulence genes excluding their affiliation to the previously described pathotypes (Table 2).

**Table 2:** Characteristics summary of invasive strains of fecal origin

Origin	Type of strains	ID	Serotype	Phylogroup	Source	Virulence genes											
						*stx-1*	*stx-2*	*ipaC*	*estA*	*elt*	*tia*	*bfpA*	*eae*	*exhA*	*aggR*	*pCVD432*	*afaD*
Humans	Invasive strains	ECOR1	ON:HN	A	Human, USA	−	−	−	−	−	−	−	−	−	−	−	−
		ECOR2	ON:H32	A	Human, USA	−	−	−	−	−	−	−	−	−	−	−	−
		ECOR9	ON:NM	A	Human, Sweden	−	−	−	−	−	−	−	−	−	−	−	−
		ECOR15	O25:NM	A	Human, Sweden	−	−	−	−	−	−	−	−	−	−	−	−
		ECOR63	ON:NM	B2	Human, Sweden	−	−	−	−	−	+	−	−	−	−	−	+
		ECOR35	O1:NM	D	Human, USA	−	−	−	−	−	−	−	−	−	−	−	−
		ECOR36	O79:H25	D	Human, USA	−	−	−	−	−	−	−	−	−	−	−	−
		ECOR43	ON:HN	E	Human, Sweden	−	−	−	−	−	−	−	−	−	−	−	−
Animals		ECOR7	O85:HN	A	Orangutan, USA	−	−	−	−	−	−	−	−	−	−	−	−
		ECOR23	O86:H43	A	Elephant, USA	−	−	−	−	−	−	−	−	−	−	−	−
		ECOR45	ON:HM	B1	Pig, Indonesia	−	−	−	−	−	−	−	−	−	−	−	−
		ECOR67	O4:H43	B1	Goat, Indonesia	−	−	−	−	−	−	−	−	−	−	−	−
		ECOR69	ON:NM	B1	Celebese ape, USA	−	−	−	−	−	−	−	−	−	−	−	−
		ECOR70	O78:NM	B1	Gorilla, USA	−	−	−	−	−	+	−	−	−	−	−	+
		ECOR52	O25:H1	B2	Orangutan, USA	−	−	−	−	−	−	−	−	−	−	−	−
		ECOR57	ON:NM	B2	Gorilla, USA	−	−	−	−	−	+	−	−	−	−	−	−
		ECOR44	ON:HN	D	Cougar, USA	−	−	−	−	−	−	−	−	−	−	−	−
		ECOR46	O1:H6	D	Celebese ape, USA	−	−	−	−	−	−	−	−	−	−	−	−
Humans	Cell detaching strains	ECOR6	ON:HM	A	Human, USA	−	−	−	−	−	−	−	−	−	−	−	−
		ECOR51	O25:HN	B2	Human, USA	−	−	−	−	−	−	−	−	−	−	−	+
		ECOR53	O4:HN	B2	Human, USA	−	−	−	−	−	−	−	−	−	−	−	−
		ECOR54	O25:H1	B2	Human, USA	−	−	−	−	−	−	−	−	−	−	−	−
		ECOR59	O4:H40	B2	Human, USA	−	−	−	−	−	−	−	−	−	−	−	−

The cell-detaching strains were also tested for their expression of virulence genes associated of enteropathogenic strains. The ECOR 51 strain was shown to express the *afaD* virulence gene, showing that this strain is classified as a DAEC. The other cell-detaching strains were negative for the tested genes (Table 2).

### Survival and replication ability of the invasive strains from the ECOR collection

The 15 strains revealed as adherent and invasive and without virulence genes associated with diarrheagenic *E. coli* pathotype were tested for their ability to survive and replicate within macrophages. Among these strains, 13 were able to survive and replicate in macrophages. The percent intracellular bacteria at 1 and 24 h postinfection ranged between 114.06 and 881.36%. The ECOR 1, 2, 9, 15, 35, 36, 43, 23, 45, 67, 69, 52, 44 were thus considered as belonging to the AIEC pathotype.

The two other strains (ECOR 46 and 7) were not able to replicate inside macrophages with an index of 70.42 and 74.51% respectively (Table 3). These strains were thus not affiliated to the AIEC pathovar.

**Table 3:** Capacity of adhesion in I-407 cells and survival and replication in macrophages among the 15 invasive strains form the ECOR collection

	ECOR strains	Mean adhesion index	Mean of bacteria recovered at 1 and 24 h postinfection (%)
Invasive strains	ECOR1	197.89±29.77	387.47%±236.00%
	ECOR35	210.53±208.41	881.36%±295.06%
	ECOR52	73.26±2.38	133.33%
	ECOR2	111.58±32.75	168.72%±5.67%
	ECOR36	145.26±68.48	511.41%±70.89%
	ECOR43	82.11±20.84	114.06%±142.24%
	ECOR44	334.74±229.25	255.70%±35.56%
	ECOR45	94.74±20.84	314.08%±268.26%
	ECOR46	92.63±17.86	70.42%
	ECOR7	170.53±74.43	74.51%
	ECOR9	157.89±56.57	152.00%
	ECOR15	153.68±44.66	279.07%
	ECOR23	260.63±48.23	395.48%
	ECOR67	67.37±23.82	361.90%
	ECOR69	117.89±41.68	887.64%
Noninvasive strains	*E. coli* K12	51.58±16.38	24.59%±2.84
	*E. coli* Nissle	32.21±8.30	87.78%±131.98%
Invasive strains	AIEC LF82	166.32±20.84	220.59%
	AIEC NRG857c	54.32±4.17	194.90%±39.93

## Discussion

The ECOR collection is a collection of *E. coli* that reflects the genetic diversity of non-pathogenic *E. coli* isolated from humans and animals of diverse geographical origins [19] (Table 1). We studied the 61 strains of *E. coli* isolated from the feces of healthy individuals for an invasive phenotype. Our study allowed the demonstration of the presence of AIEC strains in feces of healthy individuals. On the other hand this also gave an insight into the prevalence of AIEC in diverse conditions. Our results showed that intestinal *E. coli* from the ECOR collection could be divided into three groups according to their phenotypes observed in vitro in I-407 cells: (i) non-invasive strains, (ii) invasive strains defined as AIEC and (iii) strains able to disrupt I-407 cell layer.

Of a total of 61 fecal *E. coli* strains, 48 were not invasive, 18 presented adherent and invasive properties and 5 were able to detach I-407 cells. Out of 18 adherent and invasive strains, 13 were able to survive and replicate inside macrophages, these strains were thus affiliated to the AIEC pathotype. Among all the fecal strains, the prevalence of AIEC was 21% (24% in the human set and 19% among animal strains). All cell-detaching strains were of human origin, which accounted for 8% of the strains among animal and human isolates (17% of all human strains tested).

The AIEC pathotype was first described in inflammatory bowel diseases patients. Several independent research groups have reported a higher prevalence of AIEC in biopsies from IBD patients compared to non-IBD controls. However, AIEC prevalence greatly varies between studies. Indeed, AIEC prevalence was shown to range between 10 and 52% in ileal biopsies of CD patients [9–12]. This decreases to 3.7% in colonic biopsies of CD patients, and AIEC has yet to be isolated from colonic biopsies of UC patients [6]. In non-IBD controls, AIEC prevalence was shown to be between 0 and 17% [9–12]. In our study, we showed for the first time that AIEC strains can also be detected in stools of healthy subjects, with a prevalence of 21%.

AIEC pathovars from the ECOR collection are genetically highly diverse as they belong to different phylogroups. Indeed, among the 13 intestinal adherent and invasive strains identified in this study, 5 belong to the phylogroup A, 3 to the phylogroup B1, 1 to the phylogroup B2, 3 to the phylogroup D and 1 to the phylogroup E. It has been shown that *E. coli* belonging to B2 and D phylogroups possess higher numbers of virulence genes than do A and B1 phylogroups. However, recently, no association was found between the phylogenetic background and the occurrence of diarrhea in calves [23]. The majority of AIEC strains isolated to date, coming from diseased human or animals, belong to the B2 phylogroup [10, 11, 15, 17, 18]. AIEC strains isolated from non-IBD controls (mainly patients suffering from rectorrhagia, hemorrhoids, irritable bowel syndrome, or diverticulitis) also mainly belong to the B2 phylogroup [10]. We showed here that AIEC strains isolated from healthy individuals, either humans or animals, mainly belong to other phylogroups.

Some studies have reported virulence determinant genes and cytotoxic profiles in *E. coli* strains from the ECOR collection [24–27]. Strains revealed to be invasive and capable of detaching I-407 cells were found to express some virulent genes such as pyelonephritis associated pili (*pap*), S-family adhesions (*sfa*), hemolysin (*hly*), long polar fimbriae (*lpf*), and secreted autotransporter toxin (*sat*). Among the 13 adherent and invasive strains, 10 are known to express at least one of these genes (ECOR 2, 35, 36, 43, 23, 45, 67, 69, 52 and 44) and among the five cell-detaching strains, three harbor at least one of these virulence determinants (ECOR 51, 53 and 54) (Table 4). Some of these genes were also found in AIEC strains isolated from biopsies of both IBD and non-IBD patients. Martinez et al. showed that of a total of 16 AIEC strains recovered from biopsies of IBD patients, 8 express *papC* gene, 2 express *sfa*, 4 express *hlya*, and all *fimH*. AIEC strains from non-IBD patients (n = 6) also express these genes, 4 were positive for *papC*, 2 for *sfa*, 2 for *hlya* and all for *fimH* [10].

**Table 4:** Virulence determinants in AIEC and cell-detaching strains from the ECOR collection

ECOR strains	Cell-detaching strains					References
	ECOR53	ECOR51	ECOR6	ECOR59	ECOR54	
Virulence determinants associated with pathogenicity islands						
Two adhesin gene clusters sfa and pap	+ pap1.2.3 sfa1.2	+ pap1.2.3 sfa1.2	−	−	+sfa1.2	Boyd et al. [25]
The kps capsule operon	+kps1.2	+ kps1.2	−	−	+ kps1.2	
The alpha-hemolysin loci hlyI and hlyII	+hly2	+hly2	−	−	+ hly2	
Haemolytic activity						
PCR detection of the hlyA gene	+ Strong haemolysis	+ Strong haemolysis	ND	ND	+ Strong haemolysis	Lai et al. [24]
Distribution of STEC-lpfAs						
Four lpfA genetic variants designated lpfAO157/OI-141, lpfAO157/OI-154, lpfAO26 and lpfAO113	−	ND	−	−	−	Toma et al. [26]
Antibiotic resistance in the ECOR collection						
Streptomycin	−	−	−	−	−	Mazel et al. [32]
Spectinomycin	−	−	−	−	−	
Sulfonamide	−	−	−	−	+	
Tetracyclin	−	−	+	−	−	
Ampicillin	−	−	−	−	−	
Kanamycin	−	−	−	−	−	
Chloramphenicol	−	−	−	−	−	
Mercuric chloride	−	−	−	−	−	
Ethidium bromide	−	−	−	−	−	

ECOR strains	AIEC phenotype													References
	ECOR43	ECOR23	ECOR36	ECOR69	ECOR52	ECOR1	ECOR2	ECOR9	ECOR15	ECOR35	ECOR45	ECOR67	ECOR44	
Virulence determinants associated with pathogenicity islands														
Two adhesin gene clusters sfa and pap	−	−	+ pap1.2.3	−	+ pap1.2.3 sfa1.2	−	+ pap1.2.3	−	−	+ pap1	−	−	+ pap1.2.3	Boyd et al. [25]
The kps capsule operon	−	−	+ kps1.2	−	+ kps1.2	−	−	−	−	+ kps1.2	−	−	+ kps1.2	
The alpha-hemolysin loci hlyI and hlyII	+ hly2	−	−	−	+ hly2	−	−	−	−	−	−	−	−	
Haemolytic activity														
PCR detection of the hlyA gene	No haemolysis	ND	ND	ND	+ Strong haemolysis	ND	ND	ND	ND	ND	ND	ND	ND	Lai et al. [24]
Distribution of STEC-lpfAs														
Four lpfA genetic variants designated lpfAO157/OI-141, lpfAO157/OI-154, lpfAO26 and lpfAO113	lpfAO26+ lpfAO157/ OI-141+	lpfAO26+ lpfAO113+	lpfAO113+	lpfAO113+	−	−	−	−	−	lpfAO113+	lpfAO26+ lpfAO113+	lpfAO26+ lpfAO113+	lpfAO113+	Toma et al. [26]
Antibiotic resistance in the ECOR collection														
Streptomycin	−	−	−	−	−	−	−	−	−	−	−	−	−	Mazel et al. [32]
Spectinomycin	−	−	−	−	−	−	−	−	−	−	−	−	−	
Sulfonamide	−	−	−	−	−	−	−	−	−	−	−	−	−	
Tetracyclin	−	−	−	−	−	−	−	−	−	−	−	−	−	
Ampicillin	−	−	−	−	−	−	−	−	−	−	−	−	−	
Kanamycin	−	−	−	−	−	−	−	−	−	−	−	−	−	
Chloramphenicol	−	−	−	−	−	−	−	−	−	−	−	−	−	
Mercuric chloride	−	−	−	−	−	−	−	−	−	−	−	−	−	
Ethidium bromide	−	−	−	−	−	−	−	−	−	−	−	−	−	

Our work showed for the first time the detection of AIEC strains in healthy animal hosts; one from a healthy orangutan, one from a healthy elephant in captivity, one from a healthy celebese ape in captivity, one from a healthy pig, one from a healthy cougar and one from a healthy goat. These results provide further support for the absence of host specificity of this pathotype [28]. Further studies analyzing the prevalence of AIEC in well-characterized diseased and healthy animals are needed in order to detect putative reservoirs of AIEC strains and to evaluate the dimension of the risk with respect to the implementation of prevention and control measures and to public health.

To conclude, this study showed a prevalence of asymptomatic intestinal carriage of invasive pathogenic *E. coli*, supporting the argument that AIEC may be considered as pathobiont [9, 16, 29]. Large-scale studies in humans (both CD and UC patients, irritable bowel disease patients and healthy individuals) are necessary for a better knowledge of AIEC distribution and are currently in progress in our laboratory. Our clinical study is consistent with these data, showing notably a high prevalence of AIEC in feces of healthy individuals (manuscript in preparation).

## Conclusions

Our study has evaluated the prevalence of AIEC in the standard reference *E. coli* collection (ECOR). Among all the strains from the ECOR collection, 21% exhibited the AIEC phenotype, 24% were from healthy human origin and 19% from different healthy animal hosts. This is of major interest from an epidemiological point of view, highlighting potential environmental niches and possible human transmission of AIEC and susceptibility in further inflammatory events in a healthy population.

## Methods

### Bacterial strains

Bacteria from the ECOR collection were kindly provided by Laurent Debarbieux (Institut Pasteur Paris) (Table 1). *E. coli* referent strains belonging to different pathogenic groups respectively ETEC (ATCC 35401), EHEC (ATCC 43895), EPEC (ATCC 43887), EAEC (ATCC 33780), EIEC (ATCC 43893), DAEC (AfaA30, kindly provided by Pr Eric Oswald, IRSD, INSERM UMR 1220, Toulouse, France) were used as positive controls in PCR genotyping; while *E. coli* K12 was used as a negative control. *E. coli* ATCC 25922 was used as a positive control for the gentamicin susceptibility assay. Bacteria were routinely grown on Luria–Bertani (LB) agar plates or in LB broth at 37 °C.

### Gentamicin susceptibility assay

All the strains were assessed for their susceptibility to gentamicin by applying the in vitro diffusion method. Agar plates were inoculated with a 0.5 McFarland bacterial suspension. Then, gentamicin discs (Oxoid, 10 μg) were placed in the inoculated media and incubated at 37 °C for 24 h. The susceptibility of the bacteria to gentamicin was determined by measurement of the diameters of the inhibition zones surrounding the discs in millimeters according EUCAST values. A control standard strain (*E. coli* ATCC 25922) for which the susceptibility to antimicrobial agents is known is included in the test to validate the assay [30].

### Cells

Intestine-407 cell lines (I-407, ATCC accession number CCL-6™) were used to determine bacterial invasiveness. I-407 cells were maintained in Basal medium Eagle (BME, Thermofisher, France) with 10% heat-inactivated fetal calf serum (Euroclone Italy) supplemented with 1% Glutamax and 1% penicillin/streptomycin (Thermofisher, France), in 5% CO_2_ atmosphere at 37 °C.

### Adhesion and invasion assays in epithelial cells Intestine-407

For AIEC identification, adhesion and invasion assays, based on gentamicin protection, were performed according to a previously described method [6, 15, 21, 22]. 24-well plates containing 4 × 10^5^ cells/well incubated for 20 h without antibiotics were infected with the *E. coli* strains at a multiplicity of infection of 10 during 3 h of incubation time. For the bacterial adhesion assays, the cell monolayers were washed with phosphate-buffered saline and then lysed with 1% Triton X-100. Adherent bacteria were quantified by plating them on LB agar. Plating was performed over a maximum period of 30 min in order to avoid bacterial lysis by Triton X-100. Adherence ability (I_ADH) was determined by calculating the mean number of bacteria per cell. For the bacterial invasion assays, cells were washed twice with phosphate buffer saline (PBS), and fresh cell culture medium containing 100 μg ml^−1^ of gentamicin was added for 1 h to kill extracellular bacteria. Cells were then washed three times with PBS and 1 ml of 1% Triton X-100 was added in each well for 5 min to lyse the eukaryotic cells. After cell lysis, the number of intracellular bacteria was determined by plating. The invasive ability was expressed as the percentage of intracellular *E. coli* recovered from the initial inoculum, taken as 100%. An isolate was considered invasive when invasion was ≥ 0.1%. Each experiment was done in triplicate. The non-invasive strains of *E. coli* K12 and *E. coli* Nissle and the invasive AIEC LF82 and AIEC NRG857c strains were used as negative and positive control in each experiment.

### Survival and replication in J774 macrophages

The macrophage-like J774A.1 cell line (ATCC TIB-67) was used as a model in *E. coli* survival and replication assays. *E. coli* isolates with known adherence and invasion properties were checked for their ability to survive and replicate inside macrophages. Briefly, macrophages were seeded at 4 × 10^5^ cells per well in two 24-well plates and incubated for 20 h. After incubation, the medium was replaced with fresh medium and bacteria were seeded at a multiplicity of infection of 100. To promote internalization of bacteria by the macrophages, the samples were centrifuged at 1000 rpm for 10 min and incubated for an additional 10 min at 37 °C with 5% CO_2_. Nonphagocytosed bacteria were killed with gentamicin. Intracellular bacteria were quantified in the same manner as described for the invasion assays after 1 and 24 h of infection. The results are expressed as the mean percentages of bacteria recovered at 1 and 24 h postinfection: I_REPL (%) = (CFU ml^−1^ at 24 h/CFU ml^−1^ at 1 h) × 100. Those strains with an I_INV of > 0.1 and an I_REPL of > 100% were classified as AIEC strains in the present study. All assays were performed in duplicate.

### Virulence genotyping by polymerase chain reaction (PCR)

DNA was extracted from bacteria by suspending one bacterial colony in 50 μl of deionized water, boiling the suspension for 10 min, and centrifuging it at 10,000×*g* for 1 min. The supernatant was then used as the DNA template for PCR.

The presence of 12 virulence genes specifically associated with diarrheagenic *E. coli* pathotypes (EPEC, ETEC, EIEC, EHEC, DAEC, EAEC), and uropathogenic *E. coli* (UPEC) was analyzed (Additional file 1: Table S1) [8, 31]. *E. coli* reference strains were used as positive controls.

Adherent/invasive bacteria able to survive and to multiply within macrophage, without expressing virulence genes associated with diarrheagenic *E. coli* pathotypes and uropathogenic *E. coli* were considered as AIEC strains.

## Additional file

**Additional file 1: Table S1.** Primers used for *E. coli* virulence genes detection.

## Abbreviations

AIEC: adherent invasive *Escherichia coli*
BME: Basal medium eagle
CD: Crohn’s disease
ECOR: *Escherichia coli* Reference Collection
FCS: Fetal calf serum
I-407: Intestine-407 cell lines
IBD: inflammatory bowel disease
IEC: intestinal epithelial cells
LB: Luria Bertani
MLEE: multilocus enzyme electrophoresis
PBS: phosphate buffer saline
PCR: polymerase chain reaction
S: susceptible
TNF: tumor necrosis factor
UC: ulcerative colitis
USA: United States of America
