# Supplementary material for: High carriage of adherent invasive E. coli in wildlife and healthy individuals
Source: Gut Pathog. 2018 Jun 14;10:23. doi: 10.1186/s13099-018-0248-7 (PMC6001069; doi:10.1186/s13099-018-0248-7)
Supplement: Supplementary file 1 — Additional file 1: Table S1. Primers used for E. coli virulence genes detection. [file 13099_2018_248_MOESM1_ESM.pdf]

Table 4:

Virulence gene name	Pathotype	Primer sequence (5'-3')	PCR product length (bp)	References
stx-1	EHEC	Fwd: GCAAAGAMGTATGTWGATTCG<br>Rev: GWGCCACTATCAATCATCAG	107	[32]
stx-2		Fwd: AATGCAAATCAGTCGTCAC<br>Rev: TGCATCTCTGGTCATTGTAT	82	[32]
ipaC	EIEC	Fwd: CCTCACCACAAACTAACTCTAGCA<br>Rev: TGCTGCGCAGAAAGGGTATT	175	[This study]
estA	ETEC	Fwd: CACCTTTCGCTCAGGATGCT<br>Rev: TTCATGCTTTCAGGACTACTTTCATT	115	[This study]
elt		Fwd: GGYAAAAGAGAAATGGTTAT<br>Rev: TCTCGGTCAGATATGYGATTC	142	[32]
tia	ETEC /UPEC	Fwd: GGGTTATGCACGGGTACATCA<br>Rev: GCCAGATTCATTCCAGGAGGTA	61	[This study]
bfpA	EPEC	Fwd: CMGGTGTGATGTTTTACTAC<br>Rev: TGCCCAATATACARACCAT	109	[32]
eae	EPEC / EHEC	Fwd: GCTATAACRTCTTCATTGATC<br>Rev: RCTACTTTTRAAATAGTCTCG	92	[32]
ehxA		Fwd: GCACCACAACTTGAYAAACT<br>Rev: CCAGATTATTACCTACATTYTCAG	86	[32]
aggR	EAEC	Fwd: TTTATCGCAATCAGATTAARC<br>Rev: GGACAACTRCAAGCATCTAC	94	[32]
pCVD432		Fwd: AGACTCTGGCGAAAGACTGTATC<br>Rev: ATGGCTGTCTGTAATAGATGAGAAC	194	[8]
afaD	DAEC	Fwd: CGCACACGGGTTTTCATGT<br>Rev: CCCCCTTCCCGCCTTCT	153	[This study]
